# Evidence of sustained reductions in the relative risk of acute hepatitis B and C virus infections, and the increasing burden of hepatitis a virus infection in Egypt: comparison of sentinel acute viral hepatitis surveillance results, 2001–17

**DOI:** 10.1186/s12879-019-3806-9

**Published:** 2019-02-14

**Authors:** Maha Talaat, Salma Afifi, Erik J. Reaves, Hanaa Abu Elsood, Amany El-Gohary, Samir Refaey, Radi Hammad, Mostafa Abdel Fadeel, Amr Kandeel

**Affiliations:** 1 Division of Global Health Protection, Center for Global Health, U.S. Centers for Disease Control and Prevention, Cairo, Egypt; 2 U.S. Naval Medical Research Unit No. 3, Cairo, Egypt; 3 Ministry of Health and Population, Cairo, Egypt

**Keywords:** Viral hepatitis, Hepatitis a, Hepatitis B, Hepatitis C, Egypt

## Abstract

**Background:**

Egypt ranks fifth for the burden of viral hepatitis worldwide. As part of Egypt’s renewed national strategy for the elimination of viral hepatitis, surveillance for acute viral hepatitis (AVH) was re-established during 2014–2017 to describe the current epidemiology and associated risk factors, and changes from surveillance conducted during 2001–2004.

**Methods:**

Patients with suspected AVH were enrolled, completed a questionnaire, and provided blood for testing for hepatitis viruses A (HAV), B (HBV), C (HCV), D, and E (HEV) infections by enzyme-linked immunosorbent assay. Odds ratios and Chi^2^ were used to detect differences between hepatitis types by patient characteristics and exposures. Newcombe-Wilson method was used to compare results between surveillance periods 2001–2004 and 2014–2017.

**Results:**

Between 2014 and 2017, among 9321 patients enrolled, 8362 (89.7%) had one or more markers of AVH including 7806 (93.4%) HAV, 252 (3.0%) HCV, 238 (2.8%) HBV, and 31 (0.4%) HEV infection. HAV infection occurred most commonly among children < 16 years age, while HBV infection occurred among ages 16–35 years and HCV infection in ages greater than 45 years. Healthcare-associated exposures were significantly associated with HBV and HCV infections compared to HAV infection including receiving therapeutic injections, surgery, wound suture, or urinary catheter and IV line insertions, while significant lifestyle exposures included exposure to blood outside the healthcare system, IV drug use, or incarceration. Exposures significantly associated with HAV infection were attending nursery or pre-school, contact with person attending nursery or pre-school, having meals outside the home, or contact with HAV case. Compared with AVH surveillance during 2001–2004, there was a significant increase in the proportion of HAV infections from 40.2 to 89.7% (RR = 2.3) with corresponding reductions in the proportions of HBV and HCV infections from 30.0 to 2.8% (RR = 0.1) and 29.8 to 3.0% (RR = 0.1), respectively.

**Conclusions:**

Healthcare-associated exposures were significantly association with and remain the greatest risk for HBV and HCV infections in Egypt. Additional studies to evaluate factors associated with the reductions in HBV and HCV infections, and cost effectiveness of routine HAV immunization might help Egypt guide and evaluate control measures.

## Background

Globally in 2015, viral hepatitis caused 1.34 million deaths primarily from cirrhosis (720,000 deaths) and hepatocellular carcinoma (470,000 deaths) secondary to chronic hepatitis B virus (HBV) and hepatitis C virus (HCV) infections [1]. An estimated 257 million people were living with HBV infection and 71 million people with HCV infection in 2015 [1]. Hepatitis A virus (HAV) infection only causes acute hepatitis, and while there is a safe and effective vaccine, there are an estimated 120 million infections globally each year. In 2015, there were approximately 11,000 deaths worldwide, representing 0.8% of deaths from viral hepatitis primarily from fulminant disease. Annually, there are an estimated 20 million hepatitis E virus (HEV) infections and 3.3 million symptomatic cases of acute HEV. In 2015, there were 44,000 deaths from HEV infections, representing 3.3% of deaths from viral hepatitis worldwide [1].

In the Eastern Mediterranean Region, studies indicate that more than 75% of cirrhosis and hepatocellular carcinoma are attributable to HBV or HCV infections**.** The World Health Organization (WHO) estimates that 4.3 million individuals are infected with HBV and 800,000 infected with HCV in the Eastern Mediterranean Region each year [2]. Despite the availability of effective prevention strategies, HBV and HCV transmission still occurs throughout the Eastern Mediterranean Region with many of these infections acquired in healthcare setting [2]. In addition, most of the countries in this region have a high endemicity of HAV infection [3].

Viral hepatitis is one of the most significant public health problems in Egypt, with an estimated 8–10 million persons living with the disease and millions more at risk for infection [4]. Egypt ranks fifth among all countries worldwide for the burden of viral hepatitis [5]. Patients living with viral hepatitis are at greater risk for liver cirrhosis and cancer with an upsurge in cases predicted in Egypt for years to come [6]. Liver disease is a top cause of mortality in Egypt, which poses significant medical and economic burden on the country. HCV transmission is ongoing in Egypt, with incidence rates estimated at 2.4 per 1000 person-years (165,000 new infections annually) [7]. Estimates for prevalence reported from the 2008 Egypt National Demographic and Health Survey (EDHS) indicated that anti-HCV prevalence was 14.7% and HCV-RNA was 9.8 [8]. Egypt is considered to have an intermediate endemicity level of anti-HAV Ig sero-prevalence [9]. In a study conducted in 2008, it was found that the overall prevalence of anti-HAV Ig was 61.4% among children aged 2.5–18 years [10].

Given the high burden of viral hepatitis in Egypt, the Egypt Ministry of Health and Population (MoHP) established a national committee to develop a National Control Strategy for Viral Hepatitis (2014–2018) that called for effective surveillance, enhancements in prevention of HBV and HCV infections, and expanded access to care and treatment for chronically infected patients [11]. Implementation of this strategy has resulted in reductions in the prevalence of HCV-RNA from 9.8 to 7% between the 2008 and 2015 EDHS and prevalence of antibodies to hepatitis C virus (anti-HCV) from 14.7 to 10% among ages 15–59 years in Egypt [12, 13]. In addition, the prevalence of hepatitis B surface antigen (HBsAg) was estimated to be 0.8% in Egypt among ages 1–59 years [11]in the same national survey. Despite these reductions in the burden of viral hepatitis, it remains one of the most significant public health problems facing Egypt.

Egypt MoHP has a long history of conducting periodic sentinel surveillance for AVH where surveillance was most recently conducted between 2001 and 2004 [14]. The objectives of this study were to institute sentinel surveillance of AVH in Egypt to identify and characterize the causative agents, including HAV, HBV, HCV, hepatitis D virus (HDV), and HEV; identify risk factors associated with new infections; and describe the changes in the epidemiology and burden of AVH from the sentinel surveillance of AVH conducted in 2001–2004.

## Methods

### Surveillance sites

**Fig. 1:**
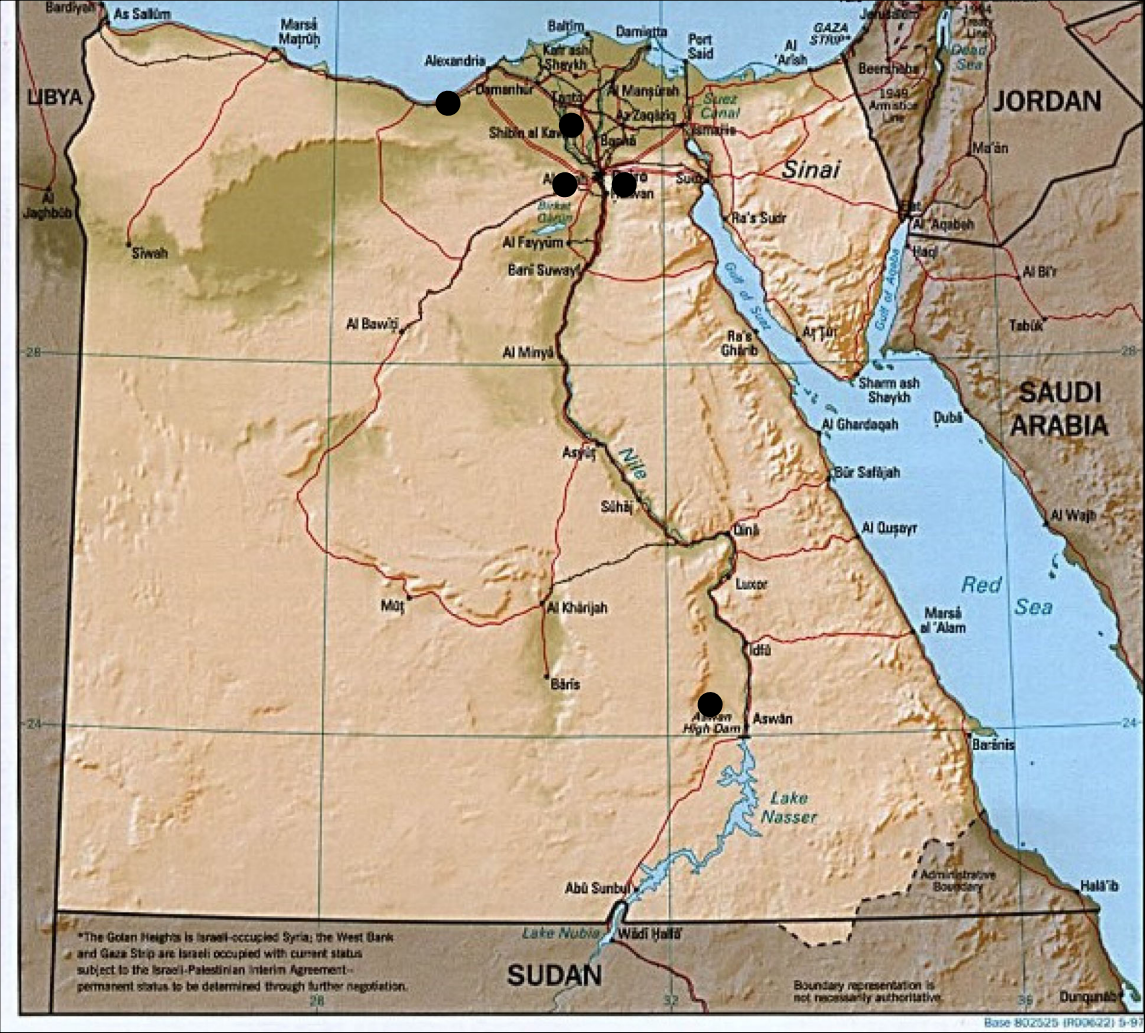
Sentinel surveillance sites for acute viral hepatitis, Egypt, January 2014–June 2017

Between January 2014 and June 2017, sentinel surveillance for AVH was conducted in a network of five infectious disease hospitals (Abbasia, Alexandria, Helwan, Menouf, and Aswan) representing the different Egyptian geographic regions (Fig. 1). Abbasia, Alexandria, and Helwan hospitals represented the main urban regions, while Menouf and Aswan represented the rural regions. Participating hospitals were selected by the MoHP based on geographic and population representation; hospital laboratory capacity to perform routine blood chemistry including alanine transaminase (ALT), aspartate transaminase (AST), and testing for viral hepatitis markers; and capacity for data management.

### Case definitions

All patients who presented to either the outpatient care unit of, or admitted to, a participating hospital were screened by hospital surveillance coordinators to ascertain whether they met the definition of a suspected case of AVH. A suspected case of AVH was defined as any patient over 12 months old who presented with sudden onset of clinical manifestations of acute hepatitis (e.g., jaundice, dark urine, fatigue, fever, nausea, vomiting, or abdominal pain), and elevated ALT ≥3 times normal limits (≥ 120 IU/L), and had no other identifiable cause of jaundice nor evidence or history of chronic liver disease.

A confirmed case of AVH was defined as a suspected case with any of the following laboratory results by enzyme-linked immunosorbent assay (ELISA) [15]:

- Acute hepatitis A: positive IgM antibody to hepatitis A virus (anti-HAV);
- Acute hepatitis B: positive IgM antibody to hepatitis B core antigen (anti-HBc);
- Acute hepatitis E: negative anti-HAV IgM and anti-HBc IgM, and positive IgM antibody to hepatitis E virus (anti-HEV);
- Acute hepatitis C: negative anti-HAV IgM, anti-HBc IgM, and anti-HEV IgM, and positive anti-HCV;
- Hepatitis D coinfection: positive anti-HBc IgM or positive IgM antibody to anti-HBsAg, and positive IgM antibody to hepatitis D virus (anti-HDV);
- Mixed hepatitis A and hepatitis B: positive anti-HAV IgM and anti-HBc IgM.

If all hepatitis markers tested negative, then patients were diagnosed as unspecified acute hepatitis [16].

### Laboratory testing and algorithm

Approximately 3–5 ml of whole blood was collected from consented patients with suspected AVH and put into red topped vacutainers. Specimens were centrifuged for 15–20 min and the serum divided into two aliquots, 0.75–1 ml each. One cryovial was initially refrigerated and later tested by ELISA for viral markers within 7–10 days, while the other cryovial was stored at − 20 °C for repeat and quality control testing. Sample preparation and ELISA serology testing were performed at the participating hospital laboratories using commercially available kits according to manufacturer’s instructions for HAV, HBV, HDV, and HEV (DIA.PRO Diagnostics Bioprobes S.r.l., Italy) and for HCV (Ortho-Clinical Diagnostics. Inc., USA).

Blood specimens of patients with suspected AVH were tested by ELISA in the participating MoHP infectious disease hospitals according to an algorithm set by the central public health laboratory (CPHL) of the MoHP. Specimens were initially tested for anti-HAV IgM, anti-HBc IgM and HBsAg. If either anti-HBc IgM or HBsAg were positive, anti-HDV IgM ELISA was performed. If either anti-HAV IgM, anti-HBc IgM, or anti-HDV IgM were positive, the diagnosis was made according to the respective results. If initial results were negative for both anti-HAV IgM and HBV markers, ELISA for anti-HEV IgM and HCV antibodies was performed.

A laboratory quality control program was developed by CPHL in collaboration with the laboratories of the U.S. Naval Medical Research Unit No. 3 (NAMRU-3) in Cairo, Egypt. This included retesting of 5% of HAV, 10% of HBV and HCV, and all HDV and HEV positive samples. A total of 10% of randomly selected negative samples were retested for confirmation of results.

### Data collection and analysis

All patients meeting the suspected case definition were asked to provide informed consent and interviewed using the same MoHP standardized case report form used for the 2001–2004 AVH surveillance period that included information on demographic characteristics and exposures to the various risk factors associated with transmission of AVH (i.e., healthcare related, lifestyle, and fecal-oral exposures) during the six months prior to the onset of symptoms. Data were entered online using a Microsoft Access data screen designed for this surveillance.

We estimated the proportion of laboratory-confirmed viral pathogens among suspected cases of acute hepatitis and calculated Risk Ratios and 95% confidence intervals to detect differences in these proportions by patient and exposure characteristics and hepatitis types. We used HAV as the reference to compare to the other hepatitis types and *p* < 0.05 to indicate statistical significance. The collection and analysis of surveillance data was supported by a NAMRU-3 Institutional Review Board approved protocol (DOD# NAMRU3.2013.0005).

### Comparing epidemiology of AVH trends over time

To describe the trends in the epidemiology of AVH over time, we compared the patient demographics, common risk factors, and proportions of the different types of AVH from our current surveillance data 2014–2017 with the previous surveillance data collected during 2001–2004. To ensure comparability, the same case definitions, laboratory methods, and common risk factors with 6-month exposure duration before symptom onset were used in the two surveillance periods. All the surveillance sites selected for the 2014–2017 surveillance period were from the same geographic regions used during the 2001–2004 surveillance period, with three of the same hospitals used during both surveillance periods (Abassia, Alexandria, and Aswan). Statistical comparison was done using Newcombe-Wilson method without continuity correction for calculating the relative risk and confidence interval for the difference between two proportions [17].

## Results

Between January 2014 and June 2017, a total of 9321 patients who met the suspected case definition of AVH were enrolled and evaluated. Their mean age was 13.6 years (range 1–90 years), 58.7% were males, 83.1% resided in urban governorates, 12.1% were unable to read or write (illiterate), and 83.5% had history of completing HBV vaccination.

Overall, 8362 (89.7%) patients had a positive test by ELISA for one or more viral hepatitis markers. Their mean age was 11.4 years (range 1–90), 59.3% were males, 83.1% resided in urban regions, 12.1% were illiterate, and 83.5% had history of completing HBV vaccination. Patients with laboratory-confirmed viral hepatitis of any type were significantly younger than patients with unspecified acute hepatitis (11.4 ± 10 vs 33.2 ± 20 years, *p* < 0.001), and were more likely to be male (59.3 vs 53.7, *p* < 0.001), illiterate (33.8 vs 14.2, *p* < 0.001), and be vaccinated for HBV (88.5 vs 39.9, *p* < 0.001).

Among patients with laboratory-confirmed disease, 7806 (93.4%) had evidence of HAV infection, 252 (3.0%) HCV infection, 238 (2.8%) HBV infection, and 31 (0.4%) HEV infection. In addition, 35 (0.4%) patients had evidence of HAV and HBV mixed infection, and 8 (3.4%) patients among those with HBV infection had evidence of coinfection with HDV (Table 1).

**Table 1:** Acute viral hepatitis cases by type of viral hepatitis and year, Egypt, January 2014–June 2017

	Total 2014–2017		2014		2015		2016		2017		Change between 2014 and 2017	
											RR	95% CI
No. of suspected AVH cases	9321		3069		2472		2859		921		NA	NA
No. of confirmed cases (% from suspected)	8362 (89.7%)		2714 (88.4%)		2210 (89.4%)		2586 (90.5%)		852 (92.5%)		1.6	1.2–2.1
Type of Hepatitis	No.	%	No.	%	No.	%	No.	%	No.	%		
HAV	7806	93.4	2505	92.3	2049	92.7	2434	94.1	818	96.0	1.8	1.4–2.2
HCV	252	3.0	106	3.9	73	3.3	60	2.3	13	1.5	0.4	0.2–0.7
HBV	238	2.8	85	3.2	70	3.2	67	2.6	16	1.9	0.6	0.4–1.1
* HDV co-infection	8	3.4	5	2.1	2	0.8	1	0.4	0	0.0	NA	NA
HAV and HBV mixed infection	35	0.4	12	0.4	11	0.5	12	0.5	0	0.0	NA	NA
HEV	31	0.4	6	0.2	7	0.3	13	0.5	5	0.6	2.8	0.8–9.1

*HAV* hepatitis A virus, *HBV* hepatitis B virus, *HCV* hepatitis C virus, *HDV* hepatitis D virus, *HEV* hepatitis E virus

*Cases of HDV coinfection are included in the frequency of HBV cases. Percent of HDV coinfection is calculated out of HBV cases (*n* = 238)

**Fig. 2:**
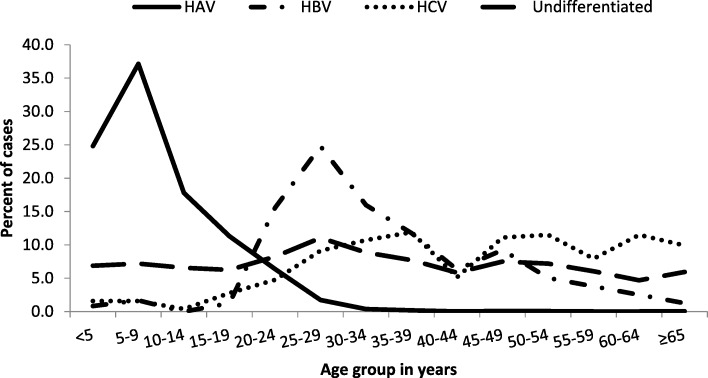
Distribution of acute viral cases by age group, Egypt, January 2014–June 2017 (*N* = 9255)

The proportion of HAV infections among confirmed cases was highest in Helwan (96.9%) and Menouf (95.4%) hospitals, while the proportion of HBV infections was highest in Abbasia (5.8%) and Aswan (4.3%) hospitals, and the proportion of HCV infection was highest in Alexandria (4.2%) and Abbasia (3.8%) hospitals. Mixed HAV and HBV infection was identified in all participating hospital surveillance sites. No HEV infections were reported from Abbasia hospital (Fig. 2).

**Fig. 3:**
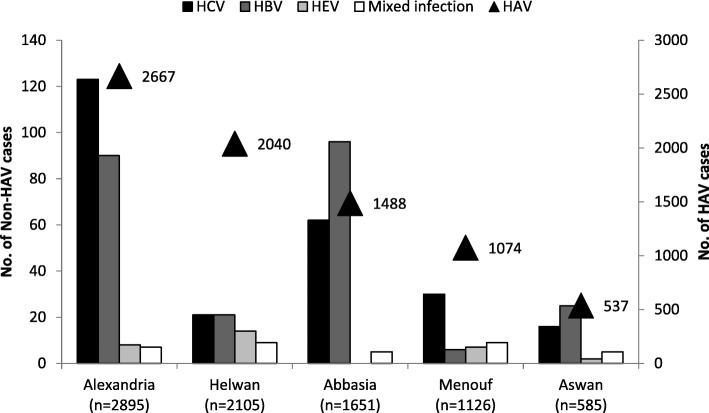
Distribution of acute viral hepatitis cases by virus type, Egypt, January 2014–June 2017

HAV infection occurred most commonly among children < 16 years of age (6388/7806; 81.8%), and the majority of patients with HAV infection were residents of urban areas (6465/7806; 82.8%). The median age of patients with HAV infection was 8 years [IQR 5–13 years] and was significantly lower in rural compared to urban areas (5 vs 8 years, *p* < 0.001) (Table 2). HBV infection occurred most commonly among persons 16–35 years of age (141/238; 59.2%), and HCV infection among persons > 45 years of age (122/252; 48.4%) (Fig. 3).

**Table 2:** Exposures related to acute viral and unspecified hepatitis, Egypt, January 2014–June 2017

	No. exposed	Total cases	Percent (%)	Odds Ratio	95% confidence interval	*p* value
a. Healthcare-associated exposures						
1. Received therapeutic injection*						
HAV	1939	7800	24.9%	Ref	Ref	Ref
HBV	72	203	35.5%	1.7	1.2–2.3	< 0.001
HCV	90	233	38.6%	1.9	1.5–2.5	< 0.001
Unspecified acute hepatitis	309	945	32.7%	1.5	1.3–1.7	< 0.001
Total	2410	9181	30.9%	NA	NA	NA
2. Wound suture						
HAV	105	7806	1.3%	Ref	Ref	Ref
HBV	7	238	2.9%	2.2	1.0–4.8	0.05
HCV	11	252	4.4%	3.3	1.8–6.3	< 0.001
Unspecified acute hepatitis	22	959	2.3%	1.7	1.1–2.7	0.02
Total	145	9255	1.6%	NA	NA	NA
3. Blood transfusion						
HAV	7	7806	0.1%	Ref	Ref	Ref
HBV	2	238	0.8%	9.4	2.0–45.7	0.03
HCV	5	252	2.0%	22.6	7.1–71.6	< 0.001
Unspecified acute hepatitis	12	959	1.3%	14.1	5.5–35.9	< 0.001
Total	26	9255	0.3%	NA	NA	NA
4. Insertion of urinary catheter						
HAV	14	7806	0.2%	Ref	Ref	Ref
HBV	7	238	2.9%	16.9	6.7–42.2	< 0.001
HCV	3	252	1.2%	6.7	1.9–23.5	0.01
Unspecified acute hepatitis	28	959	2.9%	16.7	8.8–32.8	< 0.001
Total	52	9255	0.6%	NA	NA	NA
5. Insertion of IV line						
HAV	172	7806	2.2%	Ref	Ref	Ref
HBV	21	238	8.8%	4.3	2.7–6.9	< 0.001
HCV	26	252	10.3%	5.1	3.3–7.9	< 0.001
Unspecified acute hepatitis	84	959	8.8%	4.3	3.2–5.6	< 0.001
Total	303	9255	3.3%	NA	NA	NA
6. Surgery						
HAV	110	7806	1.4%	Ref	Ref	Ref
HBV	10	238	4.2%	3.1	1.6–5.9	< 0.01
HCV	13	252	5.2%	3.8	2.0–6.7	< 0.001
Unspecified acute hepatitis	47	959	4.9%	3.6	2.5–5.1	< 0.001
Total	180	9255	1.9%	NA	NA	NA
7. Dental procedures						
HAV	492	7806	6.3%	Ref	Ref	Ref
HBV	17	238	7.1%	1.1	0.7–1.9	0.3
HCV	23	252	9.1%	1.5	0.9–2.3	0.05
Unspecified acute hepatitis	74	959	7.7%	1.2	0.9–1.6	0.05
Total	606	9255	6.5%	NA	NA	NA
b. Lifestyle exposures						
1. Exposure to blood outside health facilities						
HAV	33	7806	0.4%	Ref	Ref	Ref
HBV	11	238	4.6%	11.4	5.7–22.9	< 0.001
HCV	6	252	2.4%	5.7	2.4–13.8	< 0.01
Unspecified acute hepatitis	12	959	1.3%	3	1.5–6.0	< 0.01
Total	62	9255	0.7%	NA	NA	NA
2. Being imprisoned in last 6 months						
HAV	12	7806	0.2%	Ref	Ref	Ref
HBV	5	238	2.1%	13.9	4.9–39.9	< 0.001
HCV	4	252	1.6%	10.5	3.4–32.7	< 0.001
Unspecified acute hepatitis	3	959	0.3%	2.0	0.6–7.2	0.2
Total	24	9255	0.3%	NA	NA	NA
3. IV drug use						
HAV	6	7806	0.1%	Ref	Ref	Ref
HBV	35	238	14.7%	224.1	39.2–538.8	< 0.001
HCV	19	252	7.5%	106	41.9–267.8	< 0.001
Unspecified acute hepatitis	14	959	1.5%	19.3	7.4–50.2	< 0.001
Total	74	9255	0.8%	NA	NA	NA
4. Share tooth brushes						
HAV	118	7806	1.5%	Ref	Ref	Ref
HBV	3	238	1.3%	0.8	0.3–2.6	0.5
HCV	3	252	1.2%	0.8	0.2–2.5	0.5
Unspecified acute hepatitis	6	959	0.6%	0.4	0.2–0.9	0.01
Total	130	9255	1.4%	NA	NA	NA
5. Share razors (for males)						
HAV	39	4541	0.9%	Ref	Ref	Ref
HBV	9	183	4.9%	6	2.8–12.5	< 0.001
HCV	3	191	1.6%	1.8	0.6–6.0	0.2
Unspecified acute hepatitis	14	514	2.7%	3.2	1.7–6.0	< 0.001
Total	65	5429	1.2%	NA	NA	NA
6. At military service (for males)						
HAV	17	4541	0.4%	Ref	Ref	Ref
HBV	5	183	2.7%	7.5	2.7–20.5	0.001
HCV	0	191	0.0%	NA	NA	NA
Unspecified acute hepatitis	2	514	0.4%	1	0.2–4.5	0.4
Total	24	5429	0.4%	NA	NA	NA
7. Labor or abortion (for females)						
HAV	12	3265	0.4%	Ref	Ref	Ref
HBV	5	55	9.1%	27.1	9.2–79.8	< 0.001
HCV	2	61	3.3%	9.2	2.0–42.0	0.01
Unspecified acute hepatitis	36	445	8.1%	23.8	12.3–46.2	< 0.001
Total	55	3826	1.4%	NA	NA	NA
c. Fecal-oral exposures						
1. Attending nursery						
HAV	1368	7806	17.5%			
Non-HAV^α^	38	1449	2.6%	7.9	5.8–10.9	< 0.001
Total	1406	9255	15.2%			
2. Contact with person attending nursery						
HAV	1780	7806	22.8%			
Non-HAV^α^	209	1449	14.4%	1.8	1.5–2.0	< 0.001
Total	1989	9255	21.5%			
3. Contact with a case of HAV						
HAV	617	7806	7.9%			
Non-HAV^α^	25	1449	1.7%	4.9	3.3–7.3	< 0.001
Total	642	9255	6.9%			
4. Eat meals outside home						
HAV	6514	7806	83.4%			
Non-HAV^α^	1055	1449	72.8%	1.9	1.7–2.1	< 0.001
Total	7569	9255	81.8%			
5. Animal exposure						
HAV	850	7806	10.9%			
Non-HAV^α^	128	1449	8.8%	1.3	1.0–1.5	0.02
Total	978	9255	10.6%			

* Therapeutic injection excluding 74 patients who reported receiving illegal drugs by injection

α Non-HAV include all cases of HBV, HCV, and unspecified acute hepatitis

During the period 2014–2017, overall the proportion of HAV infections increased from 92.3 to 96.0% (RR = 1.1, CI 1.05–1.12), while HCV infections decreased from 3.9 to 1.5% (RR = 0.4, CI 0.2–0.7) and though not statistically significant HBV infections decreased from 3.2 to 1.9% (RR = 0.6, CI 0.4–1.1) (Table 1).

The case fatality rate differed by type of viral infection, being as low as 0.1% for HAV compared to 1.3% for HBV (*p* < 0.01), 2.4% for HCV (*p* < 0.001), and 2.3% for the unspecified acute hepatitis cases (*p* < 0.001) (Table 2).

**Fig. 4:**
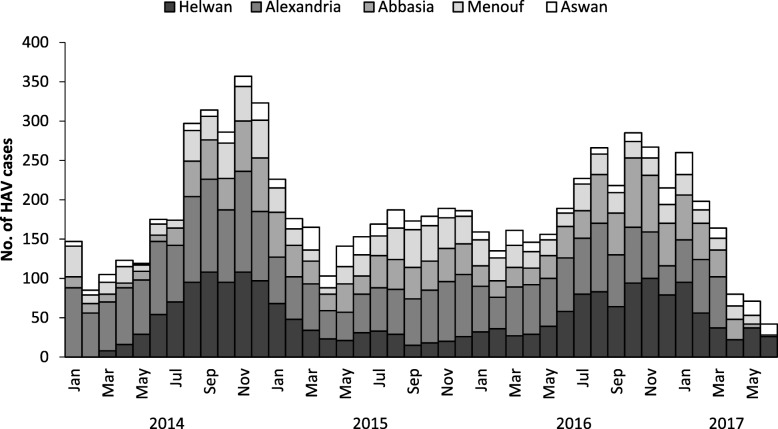
Seasonality of acute hepatitis A cases, Egypt, January 2014–June 2017

HAV infection was the main cause of AVH among the network of sentinel surveillance hospitals, occurring most commonly among children 5–19 years of age (5170; 66.2%) (Fig. 2) and residents of urban regions (6195; 79.4%) (Figs. 3). The peak seasonal distribution of HAV infection occurred between August–February corresponding to the beginning of the school year in Egypt (Fig. 4). HBV infection occurred most commonly among persons 20–44 years of age (177/238; 74.4%) with the highest proportion in Abbasia (5.8%) and Aswan (4.3%) hospitals, while HCV infection occurred most commonly in persons 20–64 years of age (211/252; 83.8%) with the highest proportion in Alexandria (4.2%) and Abbasia (3.8%) hospitals (Figs. 2 and 3). The case fatality rate was 2.4% in patients with HCV infection, 1.3% for HBV infection, and 0.1% for HAV infection.

### Risk factors

#### Healthcare-associated exposures

Receiving therapeutic injections during the past 6 months was reported by 26.2% (range 25.4–27.2%) of patients with HAV, HBV, or HCV infection or unspecified acute hepatitis. Patients with HBV or HCV infection or unspecified acute hepatitis were more likely to report having received therapeutic injections than patients with HAV infection (OR 1.7, 1.9, and 1.5, respectively; *p* < 0.001) (Table 2a). Other healthcare-associated exposures commonly associated with viral hepatitis that were recorded among patients with HBV and HCV infection and unspecified acute viral hepatitis compared to those with HAV infection included: having undergone surgery, receiving intravenous infusions, wound suture, insertion of urinary catheter and insertion of IV line (Table 2a).

#### Lifestyle exposures

Lifestyle-related exposures more strongly associated with HBV or HCV infection or unspecified acute hepatitis than HAV infection were having contact with blood outside health facilities (OR 11.4, 5.7, and 3.0, respectfully; *p* < 0.001), IV drug use (OR 244.1, 106.0, and 19.3, respectfully; *p* < 0.001) and labor or abortion among females (OR 27.1, 9.2, and 23.8; *p* < 0.001). Compared to HAV infection, being imprisoned in the last six months was more strongly associated with HBV or HCV infection (OR 13.9 and 10.5, respectfully; *p* < 0.001), and sharing a razor among males was more strongly associated with HBV infection or unspecified acute hepatitis (OR 6.0 and 3.2, respectfully; *p* < 0.001) (Table 2b).

#### Fecal-oral exposures

Compared to patients with non-HAV infection (including HBV or HCV infection, and unspecified acute hepatitis), fecal-oral exposures were more strongly associated with HAV infection and included attending a nursery or pre-school (OR 7.9; *p* < 0.001), having contact with a person attending a nursery or pre-school (OR 1.8; *p* < 0.001), having meals outside the home (OR 1.9; *p* < 0.001), or having contact with another HAV case (OR 4.9,; *p* < 0.001) (Table 2c).

### Trend of AVH epidemiology

Between surveillance periods 2001–2004 and 2014–2017, the proportion of AVH caused by HBV infection decreased from 30.0 to 2.8% (RR = 0.1, CI = 0.1–0.11)), and HCV infection decreased from 29.8 to 3.0% (RR = 0.1, CI = 0.09–0.12), while the proportion of AVH caused by HAV infection increased significantly from 40.2 to 89.7% (RR = 2.3, CI = 2.3–2.4) (Table 3).

**Table 3:** Comparison of characteristics of acute viral hepatitis cases by virus type at hepatitis sentinel surveillance infectious disease hospitals between 2001 and 2004 and 2014–2017, Egypt

Item	HAV						HBV						HCV					
	2001–2004		2014–2017		RR	95% CI	2001–2004		2014–2017		RR	95% CI	2001–2004		2014–2017		RR	95% CI
	*n*	%	*n*	%			*n*	%	*n*	%			*n*	%	*n*	%		
Viral cause	1684	40.2	8362	89.7	↑2.3	2.3–2.4	1256	30.0	238	2.8	↓0.1	0.1–0.11	1249	29.8	252	3.0	↓0.1	0.1–0.12
Male gender	1057	62.8	4540	58.2	0.9	0.89–1.0	883	70.3	183	76.9	↑1.2	1.1–1.3	847	67.8	191	75.8	1.1	1.0–1.2
Age group (years)																		
< 5	521	30.9	1936	24.8	↓↑0.8	0.7–0.9	8	0.6	2	0.8	1.3	0.3–6.2	3	0.2	4	1.6	6.6	1.5–29.3
5–19	1047	62.2	5329	68.3	1.1	1.1–1.14	232	18.5	13	5.5	↓0.3	0.2–0.5	48	3.8	14	5.6	1.6	0.8–3.0
20–44	97	5.8	522	6.7	1.2	0.9–1.4	796	63.4	182	76.5	1.2	1.0–1.3	582	46.6	112	44.4	1.0	0.8–1. 2
45–64	15	0.9	14	0.2	0.2	0.1–0.4	168	13.4	36	15.1	1.1	0.8–1.6	508	40.7	79	31.3	↑0.9	0.7–1.0
≥65	4	0.2	5	0.1	0.3	0.1–1.0	52	4.1	5	2.1	0.5	0.2–1.3	108	8.6	43	17.1	1.9	1.2–2.9
Region																		
Urban	905	53.7	6195	79.4	↑1.4	1.3–1.4	956	76.1	207	87.0	↑1.1	1.1–1.2	1095	87.7	206	81.7	0.9	0.8–1.0
Rural	779	46.3	1611	20.6	↓0.4	0.4–0.5	300	23.9	25	10.5	↓0.5	0.2–0.6	154	12.3	46	18.3	↑1.5	1.1–2.0
Risk factors																		
Received injection	278	16 .5	1939	24.8	↑1.5	1.3–1.7	352	28.0	72	30.3	1.1	0.9–1.3	443	35.5	90	35.7	1.0	0.8–1.2
Surgery	42	2.5	110	1.4	↓0.6	0.4–0.8	132	10.5	10	4.2	↓0. 4	0.2–0.7	183	14.7	13	5.2	↓0.4	0.2–0.6
Dental procedure	97	5.8	492	6.3	1.1	0.9–1.4	151	12.0	17	7.1	0.6	0.4–1.0	158	12.7	23	9.1	0.7	0.5–1.1
Blood transfusion	12	0.7	7	0.1	↓0.1	0.1–0.3	51	4.1	2	0.8	↓0.2	0.1–0.8	100	8.0	5	2.0	↓0.2	0.1–0.6
IV drug use	23	1.4	6	0.1	0.1	0.02–0.1	135	10.7	35	14.7	1.4	1.0–1.9	91	7.3	19	7.5	1.0	0.6–1.7
Household contact	111	6.6	888	11.4	↑1.7	1.4–2.1	61	4.9	6	2.5	0.5	0.2–1.2	36	2.9	6	2.4	0.8	0.4–1.9
Case fatality ratio	3	0.2	9	0.1	0.6	0.2–2.4	5	0.4	3	1.3	↑15.8	4.3–58.1	3	0.2	6	2.4	↑9.9	2.5–39.4

The comparison between surveillance periods showed a reduction in the proportion of patients infected with HAV before 5 years of age (RR = 0.8, CI = 0.7–0.9) and an increase in the proportion of patients infected at a higher age group (5–19 years) (RR = 1.1, CI = 1.1–1.14), with an increase in the number infections occurring in urban regions (RR = 1.4, CI = 1.3–1.4) and within the same household (RRs = 1.7, CI = 1.4–2.1).

Exposure to blood transfusion and to surgery within 6 months prior to infection decreased among patients with HAV, HBV, or HCV infection, while there was no significant change in the proportions of patients with viral hepatitis infections with exposure to dental procedures or IV drug use between the 2001–2004 and 2014–2017 surveillance periods (Table 3).

## Discussion

The results of this analysis indicate HAV was the main causative agent of AVH at the sentinel surveillance sites in Egypt between 2014 and 2017, representing 93.4% of the confirmed AVH cases, while HCV and HBV represented 3.0 and 2.8%, respectively. This epidemiological pattern differs significantly from AVH sentinel surveillance conducted in Egypt between 2001 and 2004, where the proportions of HAV, HBV, and HCV were 40.2, 30.0, and 29.8%, respectively [14].

Factors associated with HAV infection during this surveillance period were known risks related to fecal-oral transmission associated with unsafe water or food, and inadequate sanitation and poor personal hygiene, such as attending or being in contact with someone who attends a nursery or pre-school, eating meals outside the home, and contact with a case of acute viral hepatitis A [18, 19]. The seasonality of HAV infections reported from all hospitals in this surveillance period peaked annually during the fall and winter months (August–February) and might indicate increased infection risk associated with behaviors common during that time of year, including nursery or pre-school attendance and the start of each school year. The higher proportion of HAV infections observed in urban areas in this surveillance period compared to the previous period (83% vs 54%, *p* < 0.001) could possibly be explained by the increasing types of exposures commonly identified in urban rather than rural areas, such as attending nurseries or pre-schools and having meals outside the home where both are more readily available. In addition, the majority (> 80%) of HAV cases were reported from hospitals in three urban areas in the two largest population centers in Egypt, Cairo (Abassia and Helwan) and Alexandria (Alexandria).

The shift in the age of HAV infection from lower to higher age groups identified in this study could be explained by the fact that transitional economies in developing countries such as Egypt have variable sanitary conditions whereby children may avoid HAV infection and reach adulthood without immunity. This higher susceptibility in older age groups may lead to higher disease rates and increase in number of the outbreaks that can occur in these countries [20].Another possible explanation for the increase in the proportion of HAV infections could be an emerging HAV genotype. [21]

While we did not assess the impact of HAV infection among AVH patients, the disease can cause significant economic and social consequences, such as lengthy absences from work or school, and lost productivity. Improved sanitary conditions, food safety, and immunization are the most effective methods for preventing HAV infection [22]. Results of this surveillance indicate evidence-based control measures to ensure sanitary water and food sources and personal hygiene at nurseries, pre-schools, and food establishments might be a strategy to decrease HAV infections. Countries with intermediate endemicity, like Egypt, benefit the most from universal HAV immunization of children and should be part of a comprehensive plan to prevent and control viral hepatitis [22]. Currently, immunization for HAV is not included in the MoHP’s Expanded Program on Immunization (EPI) schedule. To determine if routine HAV immunization should be added to Egypt’s EPI schedule, additional epidemiological and economic evaluations may be necessary.

In the 17 years between the two surveillance periods, the proportion of HBV and HCV among AVH cases dropped dramatically from 30.0 to 3.0% for HBV (relative risk reduction = 93.2%) and 29.8 to 2.8% for HCV (relative risk reduction = 92.7%). These findings are consistent with the trends observed in the *Egypt Health Issues Survey 2015,* which showed a significant reduction in the prevalence of HCV among ages 15–59 years from 9.8% in 2008 to 7.0% in 2015 [13], and a 1% prevalence of active HBV infection among the Egyptian population 1–59 year of age [12]. In addition, in 2015, a substantial reduction in the incidence of HCV was described in the age groups below 20 years, reflecting lower transmission of HCV [12].

This reduction in the proportion of HBV and HCV infections among AVH could be a result of enhanced and expanded infection prevention and control (IPC) measures implemented by the MoHP in health facilities in both the public and private sectors [13, 23] and the high coverage of HBV vaccination [24]. This finding is supported in part by the reduction in the proportion of patients who reported exposure to healthcare-associated risk factors observed in the current surveillance period compared to the 2001–2004 surveillance period. The proportional reduction of HBV and HCV infections may also reflect the use of a more specific case definition, whereby the inclusion criteria used in the current surveillance period was more likely to select acute cases, or differences in population characteristics or hospital IPC practices given two of the five surveillance sites differed between surveillance periods. Operational research studies may be necessary to elucidate which strategies have helped reduce the burden of HBV and HCV infections in Egypt.

Since 2008, the national treatment program for patients with HCV infection targeted nearly one million HCV infected persons as of August 2017 (unpublished MoHP report). Between 2008 and 2016, approximately one third of the treated patients were provided pegylated interferon and ribavirin with a cure rate around 40%, and since 2016 the remaining patients were treated by the direct-acting antivirals (DAA) with a cure rate over 90% [25]. Thus, we expect a smaller reservoir of infected patients, which could have contributed to the reduction in the ongoing transmission of new HCV infections as evident by a reduced proportion of HCV cases in 2017 (1.5%) compared to previous years. Continued reduction is expected as more infected patients are treated in the future.

However, challenges remain. Despite the significant reductions in the proportion of HBV and HCV, their CFRs were higher than that reported during the previous surveillance period (1.3% vs 0.4% HBV (*p* = 0.07) and 2.4% vs 0.2% HCV (*p* < 0.001)). With the increasing availability of treatment for HCV in dedicated treatment centers in Egypt [26], the higher CFR observed could reflect more severe cases of HCV infection among persons less likely to seek treatment such as IV-drug users, or co-morbidities, as well as better case detection and laboratory diagnostics. In addition, the higher CFRs were observed primarily from one referral hospital. During this surveillance period, nearly half of those patients with HBV and HCV infection who died were IV-drug users, which is consistent with other studies reporting drug-use as one of the main causes of death in HBV and HCV patients [27, 28].

Results of this surveillance and several other studies from Egypt [29–31] suggest healthcare-associated exposures are common among patients with HBV and HCV infections. A history of receiving a therapeutic injection and insertion of an IV line were the greatest healthcare-associated exposures reported by cases of AVH and significantly associated with HBV and HCV infections in our study. Infection prevention and control programs are resource intensive and progressing rapidly in Egypt; however, expanding programs to all health care facilities could possibly minimize healthcare-related risk factors associated with viral hepatitis transmission [31].

This surveillance study was subject to some limitations. While the sentinel surveillance sites were within the largest fever hospitals where cases of AVH are referred in the largest population centers in Egypt, the data may not be representative of AVH in Egypt. The methods we used for hospital-based surveillance only identified and enrolled patients with symptomatic AVH. However, some causes of AVH, such as HCV infection, may often be asymptomatic. Defining acute HCV infection was based on anti-HCV antibody detection recommended by WHO for acute HCV surveillance systems [15]. No molecular methods to detect HCV infection were performed as recommended by the 2016 EASL26 guidelines [32] for financial constraints. Nonetheless, while data generated from hospital-based, sentinel surveillance cannot be used to estimate the burden or impact of AVH, including from asymptomatic infectious causes, it is useful to monitor disease trends over time. In addition, we compare data from this network of sentinel surveillance hospitals to data generated from a surveillance network that differed by two fever hospital sentinel sites in prior surveillance study years. These two hospitals were located in the same geographic regions shared between the surveillance periods, so we assumed the populations were comparable. And finally, we use HAV as the reference for statistical testing.

## Conclusions

In conclusion, these findings suggest significant reductions in the proportions of HBV and HCV among AVH, which is consistent with other published data, and an increase of HAV. Control measures to ensure sanitary water and food sources and personal hygiene at nurseries, pre-schools, and food establishments might be a strategy to decrease HAV infections. Additional studies may be necessary to evaluate the factors or interventions associated with the reductions in HBV and HCV infections, and cost effectiveness of routine HAV immunization in Egypt. Surveillance should be maintained for AVH and implemented for chronic HBV and HCV in Egypt to accurately identify, differentiate, and report viral hepatitis infections, and to guide and evaluate prevention programs designed to interrupt transmission.

## Abbreviations

ALT: Alanine transaminase
anti-HAV: IgM antibody to hepatitis A virus
anti-HBc: IgM antibody to hepatitis B core antigen
anti-HBsAg: IgM antibody to hepatitis B surface antigen
anti-HCV: antibody to hepatitis C virus
anti-HDV: IgM antibody to hepatitis D virus
anti-HEV: IgM antibody to hepatitis E virus
AST: Aspartate transaminase
AVH: Acute viral hepatitis
CFR: Case fatality rate
Chi2: Chi squared test
CPHL: Central public health laboratory
DAA: Direct-acting antivirals
ELISA: Enzyme-linked immunosorbent assay
EPI: Expanded Program on Immunization
HAV: Acute hepatitis A virus infection
HBV: Acute hepatitis B virus infection
HCV: Acute hepatitis C virus infection
HDV: Acute hepatitis D virus infection
HEV: Acute hepatitis E virus infection
IQR: Inter quartile range
IV: Intravenous
MoHP: Egyptian Ministry of Health and Population
NAMRU-3: U.S. Naval Medical Research Unit No. 3
